# Multimodal prognostication of autoimmune encephalitis: an Australian autoimmune encephalitis consortium study

**DOI:** 10.1007/s00415-025-13069-1

**Published:** 2025-04-25

**Authors:** Nabil Seery, Robb Wesselingh, Paul Beech, James Broadley, Sarah Griffith, Tiffany Rushen, James Beharry, Caleb Tan, Noushin Chiniforoush, Laurie McLaughlin, Liora ter Horst, Mirasol Forcadela, Tracie Tan, Christina Kazzi, Cassie Nesbitt, Katherine Buzzard, Andrew Duncan, Amy Halliday, Wendyl D’Souza, Yang Tran, Anneke Van Der Walt, Genevieve Skinner, Andrew Swayne, Charles B. Malpas, Amy Brodtmann, David Gillis, Bruce Taylor, Ernest G. Butler, Tomas Kalincik, Udaya Seneviratne, Richard Macdonell, Stefan Blum, Sudarshini Ramanathan, Stephen W. Reddel, Todd A. Hardy, Terence J. O’Brien, Paul Sanfilippo, Helmut Butzkueven, Mastura Monif, on behalf of the Australian Autoimmune Encephalitis Consortium

**Affiliations:** 1 Department of Neuroscience, Monash University, Melbourne, Victoria Australia; 2 Department of Neurology, Alfred Health, Melbourne, Victoria Australia; 3 Department of Radiology, Alfred Health, Melbourne, Victoria Australia; 4 Department of Radiology, Monash Health, Melbourne, Victoria Australia; 5 Department of Neuroscience, Monash Health, Melbourne, Victoria Australia; 6 Department of Neurology, Princess Alexandra Hospital, Brisbane, Queensland Australia; 7 School of Medicine, The University of Queensland, Brisbane, Queensland Australia; 8 Department of Neurology, Austin Health, Melbourne, Victoria Australia; 9 Department of Neuroscience, University Hospital Geelong, Geelong, Victoria Australia; 10 Department of Neurosciences, Eastern Health, Melbourne, Victoria Australia; 11 Department of Medicine, St Vincent’s Hospital, University of Melbourne, Melbourne, Victoria Australia; 12 Department of Pathology, St Vincent’s Hospital, Melbourne, Victoria Australia; 13 Department of Medicine (Royal Melbourne Hospital), University of Melbourne, Melbourne, Victoria Australia; 14 Melbourne School of Psychological Sciences, University of Melbourne, Melbourne, Victoria Australia; 15 Department of Neurology, Royal Melbourne Hospital, Melbourne, Victoria Australia; 16 Division of Immunology, Pathology Queensland Central Laboratory, Herston, Queensland Australia; 17 Department of Neurology, Royal Hobart Hospital, Hobart, Tasmania Australia; 18 Department of Neurology, Peninsula Health, Frankston, Victoria Australia; 19 CORe, Department of Medicine, The University of Melbourne, Melbourne, Victoria Australia; 20 Department of Neurology and Concord Clinical School, Concord Hospital, Concord, New South Wales Australia; 21 Translational Neuroimmunology Group, Kids Neuroscience Centre and Brain and Mind Centre, Faculty of Medicine and Health, University of Sydney, Sydney, New South Wales Australia; 22 Brain and Mind Centre, University of Sydney, Sydney, New South Wales Australia

**Keywords:** Autoimmune encephalitis, Prognostication, LGI1, Limbic encephalitis

## Abstract

**Background and objectives:**

To identify factors predictive of a favourable modified Rankin score (mRS) at 12 months in patients with autoimmune encephalitis (AE). To evaluate predictors of a binary composite clinical-functional outcome measure, encompassing mRS, drug-resistant epilepsy (DRE) and memory impairment, at 12 months.

**Methods:**

Univariable and multivariable logistic regression analyses for predictors of a favourable mRS (i.e. mRS ≤ 2) and a composite clinical-functional outcome at 12 months were used.

**Results:**

A total of 231 patients with AE were recruited. Multivariable logistic regression identified factors predictive of reduced odds of favourable mRS at 12 months were older age (OR 0.97; 95% CI 0.95, 0.98; *p* < 0.001), T2/FLAIR hyperintensity on initial MRI (OR 0.27; 95% CI 0.13, 0.56; *p* < 0.001), RSE (OR 0.17; 95% CI 0.06, 0.52; *p* = 0.002) and first-line immunotherapy failure (OR 0.18; 95% CI 0.09, 0.37; *p* < 0.001). Anti-LGI1 antibody-mediated encephalitis relative to other subtypes (OR 4.46; 95% CI 1.55, 12.80; *p* = 0.006) was associated with a better 12-month mRS. We found concordant associations for a composite outcome at 12 months, with the addition of a diagnosis of definite autoimmune limbic encephalitis (AILE) predicting a poor outcome.

**Discussion:**

Older age, MRI T2/FLAIR hyperintensity, RSE and first-line immunotherapy failure predicted worse mRS and composite clinical-functional outcome at 12 months, while a diagnosis of anti-LGI1 antibody-mediated encephalitis was associated with favourable outcomes. Our data highlight acute clinical factors predictive of a more severe clinical and functional course at 12 months.

**Supplementary Information:**

The online version contains supplementary material available at 10.1007/s00415-025-13069-1.

## Introduction

Autoimmune encephalitis (AE) comprises a heterogeneous group of immune-mediated central nervous system disorders. AE can be associated with auto-antibodies, or be antibody-negative [1]. Advances in auto-antibody characterisation and increased awareness of the disease have been associated with both increased recognition and incidence in the last 10 years. Overdiagnosis of antibody-negative disease can be problematic [2, 3]. Prognostic features are best characterised for anti-NMDAR and anti-LGI1 encephalitis [4], though recently, increased attention has been given to the study of antibody-negative disease [5], albeit limited by methodological challenges [6].

Recovery to independent living occurs in the majority of patients with antibody-mediated subtypes [4], but long-term cognitive and psychiatric sequelae, epilepsy, sleep disturbance, and persistent fatigue occur in a proportion of patients, which result in reduced productivity and quality of life [7–10]. Of these, cognitive impairment is likely the main chronic determinant of morbidity across AE subtypes [10, 11], and while less common [12], epilepsy, and drug-resistant epilepsy (DRE) in particular, represents a potentially devastating outcome [13].

Observational studies suggest that use of early immunotherapy is associated with more favourable cognitive and functional outcomes, and faster time to seizure freedom [4, 12, 14, 15]. Current treatment recommendations are largely informed by retrospective studies and expert consensus [16]. Prognostication, particularly acutely, remains challenging [17]. Moreover, the modified Rankin scale (mRS) [18] has been the most commonly used outcome measure in AE, but does not capture the full spectrum of chronic morbidity observed in patients in the “favourable” range of 0–2 mRS [7, 8]. More recently, the Clinical Assessment Scale in Autoimmune Encephalitis (CASE) has been developed and widely adopted as an AE outcome measure. This scale grades a range of relevant clinical features [19], but still does not include fatigue and executive dysfunction, which both impact functional outcome. Taken together, there is a need for a long-term outcome measure that reflects both important clinical aspects of disease severity, and functional disability.

The primary objective of the present study was to evaluate predictors of functional outcome at 12 months in a large cohort of Australian patients with AE. A secondary outcome was evaluate predictors of a binary, composite functional-clinical outcome status comprising mRS, memory impairment and drug resistant epilepsy (DRE) at 12 months.

## Methods

### Ethics approval

This study was approved by the central Human Research Ethics Committee at Alfred Health (HREC/17/Alfred/168), with a waiver of consent for medical record access for retrospectively recruited cases. All prospectively recruited cases provided informed consent.

### Patient identification

We identified adult patients with AE, aged 18 or older at time of recruitment, admitted or reviewed in neurology clinics between January 2008 and July 2023 to one of ten Australian hospitals who are part of the Australian Autoimmune Encephalitis Consortium Study: in Victoria (Alfred Health, Melbourne Health, Eastern Health, Monash Health, Austin Health, St Vincent’s Health, Barwon Health, Peninsula Health), New South Wales (Concord Repatriation General Hospital) and Queensland (Princess Alexandra Hospital). The patients were either identified through Health Information Systems (HIS) discharge codes matching the following International Coding of Disease (ICD) codes: G048, G258, G608, G049, M359, or recruited prospectively from acute neurology wards and outpatient clinics.

The minimum inclusion criteria were classification as per the 2016 position paper into the categories of ‘possible,’ ‘autoantibody-negative but probable,’ ‘definite autoimmune limbic encephalitis (AILE)’, ‘definite anti-*N*-methyl-d-aspartate receptor (NMDAR) encephalitis,’ or ‘probable anti-NMDAR encephalitis,’ in anti-NMDAR antibody-positive patients in whom cerebrospinal fluid (CSF) antibodies were either not evaluated, or no longer available in medical records [20]. The patients with other antibody-mediated encephalitides were included if compatible clinical features were accompanied by either serum or CSF autoantibodies, in lieu of established diagnostic criteria. Neural surface antibody testing was performed using indirect immunofluorescence on a commercially available fixed cell-based assay. Glial fibrillary acidic protein (GFAP) astrocytopathy testing involved detection of a suggestive neural pattern of staining using indirect immunofluorescence on commercial primate cerebrum, cerebellar and gastric tissue substrate, in lieu of availability of accredited assays in Australia. Myelin oligodendrocyte glycoprotein (MOG) antibodies (for patients with MOG cortical encephalitis) were tested using flow cytometry and live cell-based assay [21].

Patients with encephalitis associated with anti-glutamic acid decarboxylase (GAD) and onconeuronal antibodies were excluded, due to their typically disparate responsiveness to immunotherapy compared with antibody-mediated encephalitis [22, 23]. Patients with an underlying haematological malignancy and bone marrow disorders and with pre-morbid prednisolone dosing exceeding 10 mg daily were excluded from analyses involving peripheral and CSF immune cells. Patients who fulfilled the ‘possible’ AE criteria but had spontaneous recovery within 1 week were excluded and presumed to have a non-immune aetiology. Where patients presented with a predominantly new-onset refractory status epilepticus (NORSE) phenotype but otherwise met the ‘possible’ AE criteria at least, consensus as to a suspected autoimmune aetiology was required by an expert panel of neuroimmunologists (M.M, R.W) and epileptologist (T.T) for inclusion.

### Data collection

Medical records were used to collect the following data: patient demographics; acute clinical features from the time of symptom onset until discharge from the initial hospital admission, or time of first outpatient consultation; ancillary investigation results, including initial admission full blood examination (FBE), which was used to calculate neutrophil–lymphocyte (NLR) and monocyte–lymphocyte (MLR) ratios; CSF, electroencephalogram (EEG) and magnetic resonance imaging (MRI) findings (or if not admitted, initial outpatient results). Initial admission, discharge, and 12-month mRS and CASE scores were assigned by neurologists retrospectively (N.S, R.W, J.B), or prospectively during outpatient reviews. Finally, we recorded presence of relapse, and initial and long-term immunomodulatory therapy identities, doses, and start and end dates. Clinical data were collated using the Research Electronic Data Capture (REDCap) database [24, 25].

### Study definitions

The cognitive symptoms included anterograde or retrograde memory disturbance, attentional deficits and frank confusion. Consciousness impairment was defined as a Glasgow Coma Scale score of ≤ 13. Speech disturbance encompassed impairment of language and articulation (e.g. dysarthria). Psychiatric disturbance was inclusive of hallucinations or delusions, altered mood or behaviour. An associated tumour was defined as an active tumour at the time of AE diagnosis, or discovered during malignancy screening as part of AE investigations. DRE, status epilepticus (SE) and refractory SE (RSE) were defined as per ILAE definitions [26–28]. Significant memory impairment was defined as memory impairment interfering with daily activities as derived from the patient’s medical records. EEG parameters were determined from reports, with ‘discharges’ representing sharp and spike and wave and periodic discharges. A CSF pleocytosis was defined as a white blood cell (WCC) count of greater than five cells per mm^3^. MRI T2/fluid attenuated inversion recovery (FLAIR) scan hyperintensities were classified by a blinded neuroradiologist (P.B.) as AE-related if no better explanation was evident. In the absence of this, radiology reports were used for application of the same framework.

First-line immunotherapy comprised intravenous methylprednisolone (IVMP), immunoglobulin (IVIg) or plasma exchange (PLEX); second-line, rituximab or cyclophosphamide; third-line, bortezomib or tocilizumab. Failure of first-line immunotherapy was defined as either a lack of a one-point improvement in mRS within 4 weeks of initial first-line immunotherapy, or by the start date of second-line immunotherapy if this occurred before 4 weeks. Treatment delay was defined as no immunotherapy use within 4 weeks of symptom onset, including untreated patients. A relapse was defined as new or worsening clinical features following at least 2 months of stability or improvement, adapted from Titulaer et al. [4]. A “favourable” 12-month functional status was defined as a mRS score ≤ 2.

Significant long-term morbidity in individuals with AE despite a “favourable” mRS [8] is increasingly recognised, with memory impairment common across disease forms [11, 29, 30] and DRE, though less common, associated with poor quality of life [13]. We therefore, adopted a binary clinical and functional composite outcome measure, with a favourable status assigned a score of 1, and defined as the combination of mRS ≤ 2, absence of significant memory impairment, and absence of DRE. Patients who lacked any of these three features were assigned a score of 0. The patients who died were assigned the maximum CASE score of 27 (composite score of 0).

### Statistical analysis

All analyses were performed using R version 4.2.0*.* Dichotomized mRS or a composite clinical-functional outcome were used as the outcome variable for all analyses. Diagnosis variables were constructed as a binary variable of the specific diagnostic category as compared to all others (e.g. “possible” cf. other). All other independent variables were binary with exception of initial mRS and CASE scores (both integer), and peripheral blood, CSF parameters (other than CSF pleocytosis and CSF WCC > 20) and time to first line therapy (all continuous).

For analyses, missing data were handled using multiple imputation by chained equations (MICE) with predictive mean matching applied to all variables. Five imputed datasets were generated, with 10 iterations per imputation cycle. Following imputation, logistic regression models were fitted with each imputed dataset, with results pooled using Rubin’s rules. Odds ratios (OR) and 95% confidence intervals (CI) were derived from pooled estimates.

Univariable logistic regression models were constructed for predictors of 12-month favourable mRS or composite clinical-functional outcome; patients who died prior to the 12-month follow-up interval due to medical conditions not directly related to the underlying AE or associated malignancy were excluded (Table S2). Covariates with significant associations in univariable analyses (*p* < 0.05) were used to construct final multivariable logistic regression models. Given mRS and CASE are known to correlate [31], only mRS was considered for final multivariable models. RSE was favoured over SE where relevant for multivariable models given its more stringent definition. Sequential elimination of covariates with highest *p-*value until only statistically significant associations remained (*p* < 0.05) was then undertaken.

To assess for internal validity, bootstrapping with 1000 resamples was applied to each imputed dataset. For each bootstrap iteration, logistic regression was refitted to a resampled dataset, and the estimated coefficients were stored. The final results were obtained by aggregating bootstrapped estimates across imputations. Odds ratios and their confidence intervals were computed by exponentiating the bootstrapped estimates.

Sensitivity analyses were conducted to explore the robustness of the composite outcome. Three analyses involved exclusion of one component of the composite outcome respectively. A final analysis involved ordinal regression with different weighting to individual components of the composite outcome measure. All sensitivity analyses were performed using the imputed dataset.

## Results

### Baseline variables

**Fig. 1:**
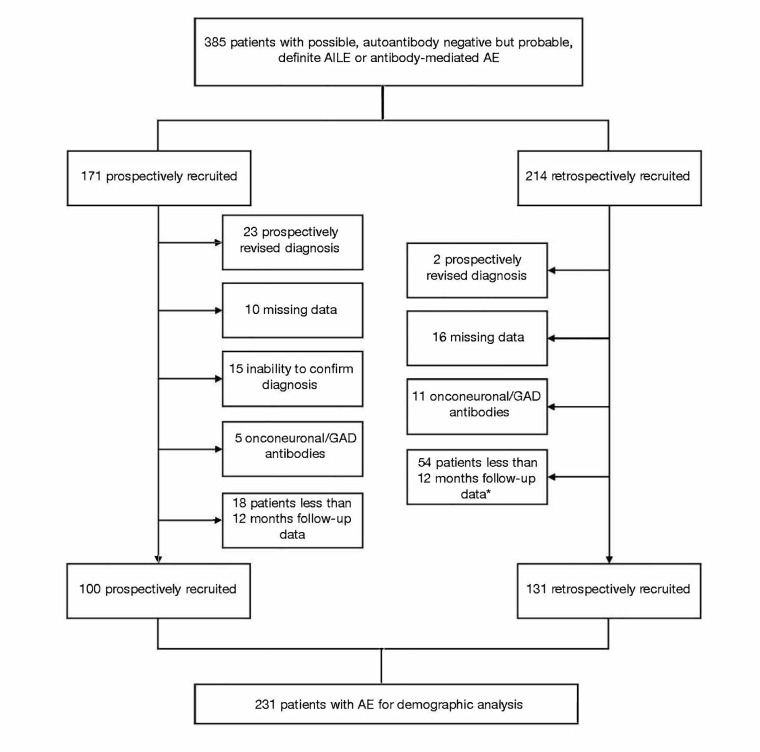
Study overview. *Including 5 patients who died prior to 12 months of an aetiology not directly related to the underlying AE or associated malignancy

We identified a total of 385 patients of whom 231 patients ultimately remained in the study (Fig. 1), and of these, a subset were previously reported [9, 32–35]. The baseline demographic and clinical characteristics are presented in Table 1. A total of 95, 11 and 16 met the ‘possible,’ ‘autoantibody negative but probable’ and ‘definite autoimmune limbic encephalitis’ criteria, respectively. A further 48 and 39 patients had diagnoses of anti-NMDAR and anti-LGI1 Ab-mediated encephalitis respectively; other antibody-mediated subtypes are presented in Table 1. Three patients met the ‘possible’ criteria in the context of immune-checkpoint inhibitors.

**Table 1:** Baseline demographics

	Overall, *N* = 231	Possible, *N* = 95	Probable, *N* = 11	Definite AILE, *N* = 16	NMDAR, *N* = 48	LGI1, *N* = 39	CASPR2, *N* = 7	GABAB, *N* = 2	AMPAR, *N* = 4	IgLON5, *N* = 1	GFAP, *N* = 3	MOG-CE, *N* = 1	ICI-E, *N* = 3
Age onset, years—median (IQR)	55.8 (34.1, 69.2)	63.2 (44.6, 71.2)	58.7 (50.3, 73.5)	44.6 (32.4, 66.6)	25.8 (22.0, 38.6)	58.3 (51.8, 71.8)	68.5 (64.4, 69.3)	52.0 (43.7, 60.4)	47.6 (34.1, 57.0)	64.4 (64.4, 64.4)	65.8 (43.7, 70.4)	34.1 (34.1, 34.1)	51.9 (48.9, 59.8)
Female (*N*, %)	122 (53%)	45 (47%)	6 (55%)	10 (62%)	37 (77%)	17 (44%)	1 (14%)	2 (100%)	1 (25%)	0 (0%)	1 (33%)	1 (100%)	0 (0%)
*Clinical features*^a^													
Cognitive^b^	211 (91%)	85 (89%)	10 (91%)	15 (94%)	44 (92%)	37 (95%)	7 (100%)	2 (100%)	4 (100%)	0 (0%)	3 (100%)	1 (100%)	2 (67%)
Seizures	152 (66%)	61 (64%)	3 (27%)	10 (62%)	31 (65%)	35 (90%)	5 (71%)	2 (100%)	1 (25%)	0 (0%)	1 (33%)	1 (100%)	1 (33%)
Hallucinations or delusions	79 (34%)	26 (27%)	0 (0%)	4 (25%)	33 (69%)	9 (23%)	3 (43%)	2 (100%)	1 (25%)	0 (0%)	0 (0%)	0 (0%)	1 (33%)
Behavioural change	102 (44%)	33 (35%)	1 (9.1%)	6 (38%)	40 (83%)	14 (36%)	3 (43%)	2 (100%)	1 (25%)	0 (0%)	0 (0%)	0 (0%)	1 (33%)
Altered mood	72 (31%)	20 (21%)	3 (27%)	5 (31%)	25 (52%)	11 (28%)	5 (71%)	1 (50%)	1 (25%)	0 (0%)	0 (0%)	0 (0%)	0 (0%)
Consciousness disturbance^c^	101 (44%)	49 (52%)	9 (82%)	6 (38%)	24 (50%)	6 (15%)	2 (29%)	2 (100%)	0 (0%)	0 (0%)	2 (67%)	0 (0%)	1 (33%)
Speech^d^	72 (31%)	30 (32%)	4 (36%)	3 (19%)	20 (42%)	8 (21%)	2 (29%)	0 (0%)	0 (0%)	1 (100%)	0 (0%)	1 (100%)	2 (67%)
Movement disorder	61 (26%)	23 (24%)	4 (36%)	2 (12%)	27 (56%)	3 (7.7%)	0 (0%)	0 (0%)	0 (0%)	0 (0%)	1 (33%)	0 (0%)	1 (33%)
SE^e^	41 (18%)	19 (20%)	3 (27%)	4 (25%)	8 (17%)	2 (5.1%)	1 (14%)	2 (100%)	0 (0%)	0 (0%)	1 (33%)	0 (0%)	1 (33%)
Refractory SE	23 (10%)	13 (14%)	2 (18%)	2 (12%)	0 (0%)	1 (2.6%)	1 (14%)	2 (100%)	0 (0%)	0 (0%)	1 (33%)	0 (0%)	1 (33%)
Acute ICU^f^	87 (38%)	42 (44%)	7 (64%)	4 (25%)	21 (44%)	7 (18%)	1 (14%)	2 (100%)	0 (0%)	0 (0%)	2 (67%)	0 (0%)	1 (33%)
Mechanical ventilation	59 (26%)	27 (28%)	7 (64%)	3 (19%)	13 (27%)	3 (8%)	1 (14%)	2 (100%)	0 (0%)	0 (0%)	2 (67%)	0 (0%)	1 (33%)
Tumour^g^	33 (14%)	9 (10%)	2 (18%)	3 (19%)	13 (27%)	0 (0%)	0 (0%)	1 (50%)	2 (50%)	0 (0%)	0 (0%)	0 (0%)	3 (100%)

All continuous variables reported as median with interquartile range

AILE, autoimmune limbic encephalitis; NMDAR, *N*-methyl-d-aspartate receptor; LGI1, leucine-rich glioma-inactivated 1; CASPR2, contactin-associated protein-like 2; AMPAR, α-amino-3-hydroxy-5-methyl-4-isoxazole propionic acid receptor; GFAP, glial fibrillary acid protein astrocytopathy; MOG CE, myelin oligodendrocyte glycoprotein cortical encephalitis; ICI-E, immune checkpoint inhibitor encephalitis

^a^Clinical features: cumulative clinical features during initial acute admission, or present up to time of initial consultation

^b^Cognitive: amnestic, attentional features or acute confusional state

^c^Consciousness disturbance: Glasgow Coma Scale score ≤ 13

^d^Speech disturbance: encompasses both language deficits and dysarthria

^e^Status epilepticus: based on ILAE definition (> 5 min tonic–clonic seizure activity OR incomplete recovery between successive tonic clonic seizures or ≥ 10 min FIAS)

^f^Acute ICU, mechanical ventilation: ICU admission and/or intubation during initial presentation

^g^Tumour: active tumour at time of AE diagnosis, or newly diagnosed during work up of AE (inclusive of tumours in all ICI-E cases)

The median age at symptom onset was 55.8 years (IQR 34.1–69.2) and 122 (53%) patients were female. SE and RSE occurred in 41 (18%) and 23 (10%) patients respectively. ICU admission was required in 87 (38%) cases, and mechanical ventilation in 59 (26%). An underlying neoplasm was present or detected in 33 (14%) patients, most frequently mature ovarian teratomas, seen in 10 (21%) of the anti-NMDAR Ab-mediated encephalitis patients; full details are presented in Table S1.

**Fig. 2:**
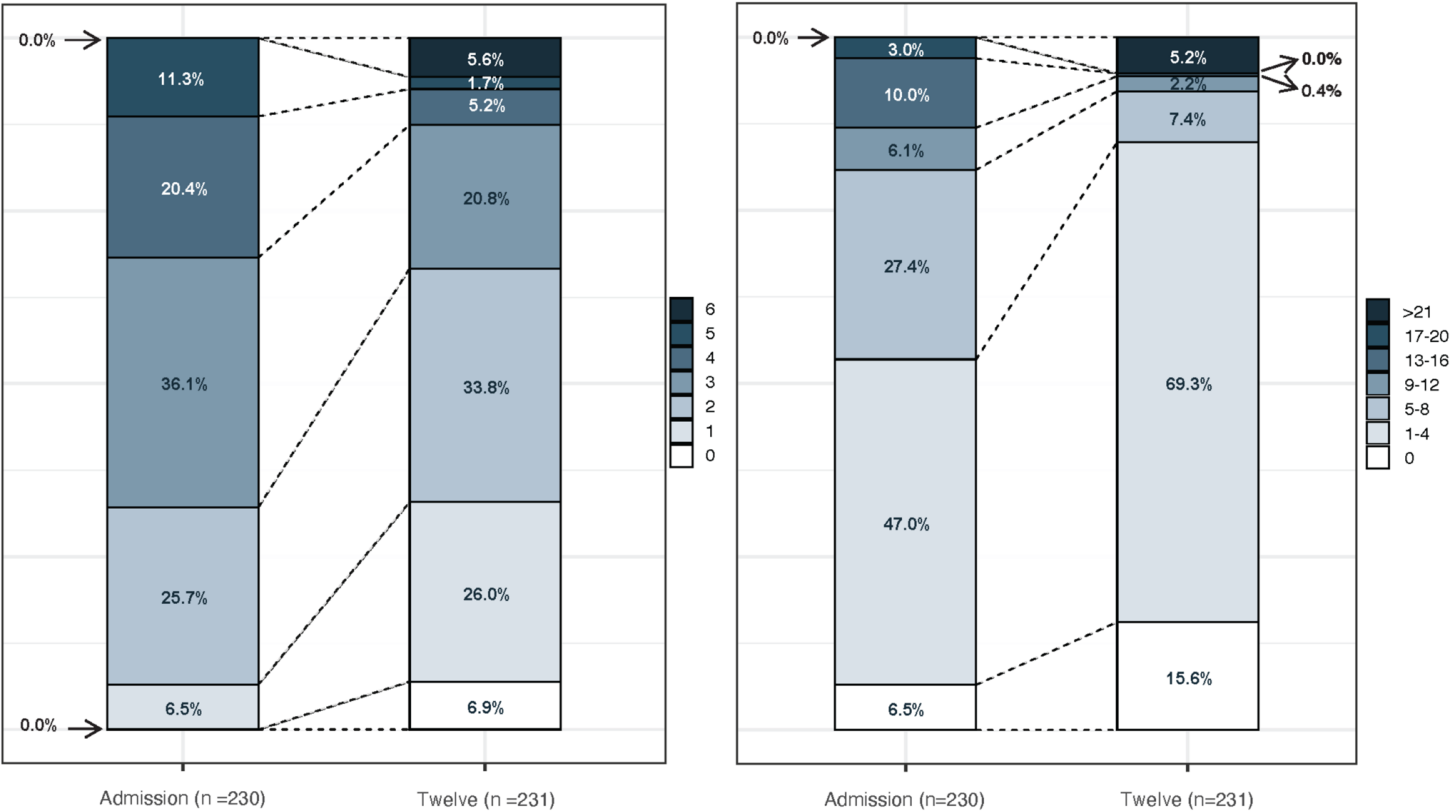
**A** mRS and **B** CASE comparisons from admission to 12 months

First-line, second-line, and third-line immunotherapy were administered in 221 (96%), 103 (45%) and 5 (2%) patients respectively, with a median time to first line therapy of 37 days (IQR 14–118) (Table 2). At admission, the median mRS and CASE scores were 3 (IQR 2–4) and 4 (IQR 2–7) respectively. At 12 months, the respective scores were 2 (IQR 1–3) and 2 (IQR 1–4). At 12 months, a favourable mRS (≤ 2) occurred in 154 (67%) patients (Fig. 2), and a favourable composite clinical-functional outcome was seen in 119 (52%). The causes of death prior to 12 months are detailed in Table S2. Other post-acute outcomes are presented in Table 2. Routine laboratory parameters, MRI and EEG findings are shown in Table 3.

**Table 2:** Treatment, hospital outcomes, sequelae

Characteristic	Overall, *N* = 231	Possible, *N* = 95	Probable, *N* = 11	Definite AILE, *N* = 16	NMDAR, *N* = 48	LGI1, *N* = 39	CASPR2, *N* = 7	GABAB, *N* = 2	AMPAR, *N* = 4	IgLON5, *N* = 1	GFAP, *N* = 3	MOG-CE, *N* = 1	ICI-E, *N* = 3
1st line^a^, (*N*, %)	221 (96%)	87 (92%)	10 (91%)	16 (100%)	47 (98%)	39 (100%)	7 (100%)	2 (100%)	4 (100%)	1 (100%)	3 (100%)	1 (100%)	3 (100%)
2nd line < 12m^b^, (*N*, %)	74 (32%)	15 (16%)	2 (18%)	8 (50%)	27 (56%)	12 (31%)	2 (29%)	2 (100%)	3 (75%)	0 (0%)	1 (33%)	0 (0%)	1 (33%)
2nd line^c^ (*N*, %)	103 (45%)	25 (26%)	2 (19%)	11 (69%)	35 (73%)	18 (46%)	5 (71%)	2 (100%)	3 (75%)	0 (0%)	1 (33%)	0 (0%)	1 (33%)
3rd line^c,d^, (*N*, %)	5 (2%)	1 (1%)	0 (0%)	0 (0%)	3 (6%)	0 (0%)	0 (0%)	0 (0%)	0 (0%)	0 (0%)	0 (0%)	0 (0%)	0 (0%)
Time to 1st line therapy (days)	37 (14, 118)	48 (12, 153)	20 (8, 40)	14 (9, 32)	30 (15, 52)	62 (28, 200)	187 (81, 352)	33 (32, 34)	28 (22, 32)		10 (9, 92)	22 (22, 22)	12 (8, 17)
Initial mRS	3 (2, 4)	3 (3, 4)	3 (3, 4)	3 (3, 4)	3 (2, 4)	2 (2, 3)	3 (2, 3)	4 (3, 4)	3 (3, 3)	2 (2, 2)	3 (2, 4)	3 (3, 3)	2 (2, 2)
12 m mRS	2 (1, 3)	2 (1, 3)	3 (2, 6)	3 (2, 3)	2 (1, 2)	2 (1, 2)	2 (2, 3)	2 (2, 3)	2 (2, 2)	2 (2, 2)	2 (2, 2)	1 (1, 1)	1 (1, 2)
12 m mRS ≤ 2	154 (67%)	62 (65%)	4 (36%)	5 (31%)	37 (77%)	32 (82%)	4 (57%)	1 (50%)	3 (75%)	1 (100%)	2 (67%)	1 (100%)	2 (67%)
12 m composite outcome favourable	119 (52%)	51 (54%)	3 (27%)	1 (6.2%)	30 (63%)	26 (67%)	1 (14%)	1 (50%)	1 (25%)	1 (100%)	1 (33%)	1 (100%)	2 (67%)
Initial CASE	4 (2, 7)	5 (3, 8)	4 (2, 9)	5 (2, 8)	4 (2, 7)	3 (2, 5)	4 (3, 5)	10 (5, 14)	2 (2, 2)	0 (0, 0)	3 (2, 8)	4 (4, 4)	2 (2, 4)
12 m CASE^e^	2 (1, 4)	2 (1, 4)	4 (2, 27)	3 (2, 4)	2 (1, 3)	2 (1, 3)	3 (2, 4)	6 (4, 8)	2 (2, 2)	0 (0, 0)	3 (2, 4)	1 (1, 1)	2 (1, 3)
Relapse < 12 m	23 (10%)	8 (8%)	0 (0%)	2 (12%)	5 (10%)	7 (18%)	0 (0%)	0 (0%)	1 (25%)	0 (0%)	0 (0%)	0 (0%)	0 (0%)
Relapse < final	54 (23%)	20 (21%)	0 (0%)	2 (13%)	14 (29%)	14 (36%)	3 (43%)	0 (0%)	1 (25%)	0 (0%)	0 (0%)	0 (0%)	0 (0%)
DRE^f^ 12 m	17 (7%)	7 (7%)	0 (0%)	5 (31%)	3 (6%)	0 (0%)	0 (0%)	1 (50%)	0 (0%)	0 (0%)	0 (0%)	0 (0%)	1 (33%)
DRE final	18 (8%)	7 (7%)	0 (0%)	5 (31%)	4 (8%)	0 (0%)	0 (0%)	1 (50%)	0 (0%)	0 (0%)	0 (0%)	0 (0%)	1 (33%)
Mortality^g^	32 (14%)	14 (15%)	5 (45%)	0 (0%)	4 (8%)	3 (8%)	0 (0%)	1 (50%)	2 (50%)	1 (100%)	0 (0%)	0 (0%)	2 (67%)

All continuous variables reported as median with interquartile range

AILE, autoimmune limbic encephalitis; NMDAR, *N*-methyl-d-aspartate receptor; LGI1, leucine-rich glioma-inactivated 1; CASPR2, contactin-associated protein-like 2; AMPAR, α-amino-3-hydroxy-5-methyl-4-isoxazole propionic acid receptor; GFAP, glial fibrillary acid protein astrocytopathy; MOG CE, myelin oligodendrocyte glycoprotein cortical encephalitis; ICI-E, immune checkpoint inhibitor encephalitis

^a^First line immunotherapy: intravenous methylprednisolone, intravenous immunoglobulin or plasma exchange

^b^Second line immunotherapy: rituximab, cyclophosphamide

^c^Administered in overall study period

^d^Third line immunotherapy: bortezomib, tocilizumab

^e^Deceased patients assigned a CASE score of 27

^f^DRE defined as failure of two adequately dosed anti-seizure medications

^g^At last follow-up

**Table 3:** Routine initial ancillary investigation parameters

Characteristic	Overall, *N* = 231	Possible, *N* = 95	Probable, *N* = 11	Definite AILE, *N* = 16	NMDAR, *N* = 48	LGI1, *N* = 39	CASPR2, *N* = 7	GABAB, *N* = 2	AMPAR, *N* = 4	IgLON5, *N* = 1	GFAP, *N* = 3	MOG-CE, *N* = 1	ICI-E, *N* = 3
WCC^a^	8.50 (6.60, 10.30)	8.66 (6.75, 10.42)	8.40 (6.80, 8.80)	8.15 (5.83, 9.73)	8.20 (6.30, 9.30)	8.20 (6.75, 10.00)	8.90 (6.45, 10.45)	10.05 (9.43, 10.68)	7.78 (5.16, 10.80)	6.60 (6.60, 6.60)	11.40 (9.15, 14.55)	4.00 (4.00, 4.00)	8.90 (7.90, 9.90)
Neutrophils	5.70 (4.00, 7.65)	5.92 (4.04, 7.99)	5.30 (3.53, 6.19)	5.25 (3.15, 7.55)	5.50 (4.10, 7.28)	5.60 (4.80, 7.22)	5.50 (3.50, 7.47)	8.07 (7.44, 8.71)	3.65 (2.60, 6.31)	4.00 (4.00, 4.00)	9.61 (7.31, 12.75)	2.40 (2.40, 2.40)	6.00 (3.95, 7.47)
Lymphocytes	1.50 (1.10, 2.07)	1.60 (1.05, 2.10)	0.80 (0.70, 1.70)	1.39 (0.90, 1.74)	1.60 (1.29, 2.03)	1.48 (1.20, 1.83)	2.18 (1.70, 2.30)	1.40 (1.35, 1.44)	2.35 (1.55, 2.93)	1.80 (1.80, 1.80)	0.85 (0.73, 0.98)	1.20 (1.20, 1.20)	1.42 (1.14, 1.76)
Monocytes	0.66 (0.50, 0.90)	0.70 (0.50, 0.93)	0.80 (0.29, 0.90)	0.70 (0.48, 0.90)	0.60 (0.50, 0.81)	0.60 (0.50, 0.80)	0.60 (0.55, 0.78)	0.45 (0.43, 0.48)	0.65 (0.33, 0.98)	0.60 (0.60, 0.60)	0.92 (0.81, 0.99)	0.30 (0.30, 0.30)	0.62 (0.55, 0.66)
NLR	3.84 (2.34, 6.15)	3.91 (2.38, 6.47)	3.88 (2.12, 7.57)	3.25 (2.20, 4.94)	3.48 (2.54, 5.58)	4.08 (2.64, 5.67)	2.39 (1.89, 3.68)	5.75 (5.49, 6.01)	3.50 (1.21, 7.49)	2.22 (2.22, 2.22)	14.45 (10.17, 15.24)	2.00 (2.00, 2.00)	2.86 (2.55, 4.58)
MLR	0.41 (0.29, 0.64)	0.41 (0.29, 0.69)	0.53 (0.38, 1.13)	0.54 (0.29, 0.64)	0.40 (0.30, 0.53)	0.44 (0.28, 0.68)	0.34 (0.26, 0.41)	0.33 (0.30, 0.36)	0.39 (0.26, 0.67)	0.33 (0.33, 0.33)	1.08 (0.86, 1.42)	0.25 (0.25, 0.25)	0.33 (0.33, 0.53)
CSF WCC^b^	4 (1, 20)	3.00 (0.00, 13)	30 (9, 66)	9 (0, 36)	16 (6, 40)	1 (0, 4)	4 (3, 6)	23 (17, 29\)	25 (3, 50)	1 (1, 1)	133 (94, 272)	1 (1, 1)	71 (37, 93)
CSF WCC > 20^b^	56 (25%)	17 (18%)	7 (64%)	5 (31%)	19 (42%)	0 (0%)	0 (0%)	1 (50%)	2 (50%)	0 (0%)	3 (100%)	0 (0%)	2 (67%)
CSF lymphocytes^b^	4 (0, 20)	2.00 (0.00, 8)	29 (8, 59)	7 (0, 36)	15 (6, 37)	0 (0, 2)	4 (2, 8)	22 (16, 27)	25 (3, 49)		130 (90, 260)	1 (1, 2)	71 (37, 108)
CSF protein	0.49 (0.34, 0.69)	0.51 (0.36, 0.73)	0.89 (0.76, 1.05)	0.54 (0.49, 0.65)	0.39 (0.28, 0.53)	0.44 (0.32, 0.53)	0.56 (0.50, 0.65)	0.42 (0.39, 0.45)	0.62 (0.47, 0.74)	1.45 (1.45, 1.45)	2.23 (1.50, 2.30)	0.22 (0.22, 0.22)	0.49 (0.41, 2.62)
CSF intrathecal OCB^c^	19 (8.2%)	5 (5.3%)	1 (9.1%)	0 (0%)	10 (21%)	2 (5.1%)	0 (0%)	0 (0%)	0 (0%)	0 (0%)	1 (33%)	0 (0%)	0 (0%)
MRI T2/FLAIR abnormalities^d^	77 (34%)	27 (29%)	7 (64%)	13 (81%)	8 (17%)	17 (44%)	1 (17%)	0 (0%)	2 (50%)	0 (NA%)	1 (33%)	0 (0%)	1 (33%)
EEG focal slowing	60 (28%)	22 (26%)	3 (27%)	8 (50%)	11 (24%)	10 (29%)	4 (57%)	0 (0%)	0 (0%)	0 (0%)	1 (50%)	0 (0%)	0 (0%)
EEG generalised slowing	92 (43%)	44 (51%)	10 (91%)	4 (25%)	20 (44%)	7 (20%)	0 (0%)	2 (100%)	1 (25%)	0 (0%)	2 (100%)	0 (0%)	3 (100%)
EEG discharges	39 (18%)	19 (22%)	2 (18%)	4 (25%)	3 (6.7%)	6 (17%)	0 (0%)	1 (50%)	1 (25%)	0 (0%)	1 (50%)	1 (100%)	2 (50%)
EEG electrographic seizures	36 (17%)	16 (19%)	1 (9.1%)	3 (19%)	5 (11%)	7 (20%)	1 (14%)	1 (50%)	0 (0%)	0 (0%)	1 (50%)	1 (100%)	0 (0%)

All continuous variables reported as median with interquartile range

AILE, autoimmune limbic encephalitis; NMDAR, *N*-methyl-d-aspartate receptor; LGI1, leucine-rich glioma-inactivated 1; CASPR2, contactin-associated protein-like 2; AMPAR, α-amino-3-hydroxy-5-methyl-4-isoxazole propionic acid receptor; GFAP, glial fibrillary acid protein astrocytopathy; MOG CE, myelin oligodendrocyte glycoprotein cortical encephalitis; ICI-E, immune checkpoint inhibitor encephalitis

^a^Peripheral cell counts × 10^9^/L

^b^Cells/mm^3^

^c^Presented as proportion of cases who had paired serum, CSF oligoclonal band testing available

^d^Abnormalities attributable to AE

### Predictors of 12-month mRS

In multivariable analysis, a diagnosis of anti-LGI1 Ab-mediated encephalitis compared to other subtypes (OR 4.46; 95% CI 1.55, 12.80; *p* = 0.006) was associated with a better 12-month mRS (mRS ≤ 2) (Tables 4 and S4). Older age (OR 0.97; 95% CI 0.95, 0.98; *p* < 0.001), associated MRI T2/FLAIR hyperintensity (OR 0.27; 95% CI 0.13, 0.56; *p* < 0.001), RSE (OR 0.17; 95% CI 0.06, 0.52; *p* = 0.002) and first-line immunotherapy failure (OR 0.12; 95% CI 0.05, 0.27; *p* < 0.001) were associated with reduced odds of the same. The bootstrapped estimates were consistent with the original model (Table S5).

**Table 4:** Multivariable logistic regression for favourable mRS at 12 m

	OR (95% CI)	*p*
Age onset	0.97 (0.95, 0.98)	< 0.001
Anti-LGI1	4.46 (1.55, 12.80)	0.006
Refractory SE	0.17 (0.06, 0.52)	0.002
MRI T2/FLAIR	0.27 (0.13, 0.56)	< 0.001
First line failure	0.18 (0.09, 0.37)	< 0.001

LGI1, leucine-rich glioma-inactivated 1; SE, status epilepticus; MRI, magnetic resonance imaging; FLAIR, fluid attenuated inversion recovery

### Predictors of 12-month composite clinical-functional outcome status

Concordant associations were observed for predictors of a composite clinical-functional status at 12 months, with anti-LGI1 Ab-mediated encephalitis predicting a favourable status, and older age, associated MRI T2/FLAIR hyperintensity, RSE and first-line immunotherapy failure predicting reduced odds of the same. In addition, a diagnosis of definite AILE compared to other subtypes (OR 0.04; 95% CI 0.005, 0.45; *p* = 0.009) was associated with reduced odds of favourable composite outcome status at 12 months (Tables 5 and S6). The bootstrapped estimates were again consistent with the original model (Table S7).

**Table 5:** Multivariable logistic regression models for favourable composite clinical-functional outcome at 12 m

	OR (95% CI)	*p*
Age onset	0.97 (0.95, 0.99)	< 0.001
AILE	0.04 (0.005, 0.45)	0.009
Anti-LGI1	2.50 (1.05, 5.95)	0.04
Refractory SE	0.08 (0.02, 0.32)	< 0.001
MRI T2/FLAIR	0.44 (0.21, 0.92)	0.04
First-line failure	0.22 (0.11, 0.43)	< 0.001

AILE, autoimmune limbic encephalitis; LGI1, leucine-rich glioma-inactivated 1; SE, status epilepticus; MRI, magnetic resonance imaging; FLAIR, fluid attenuated inversion recovery

### Sensitivity analyses exploring robustness of composite clinical-functional status outcome

Removal of the DRE or the memory impairment components of the composite outcome measure did not alter the direction or significance of association of covariates (Tables S8 and S9, respectively), and exclusion of the mRS component revealed similar findings (Table S10), with only one covariate no longer significantly associated with 12-month outcome (anti-LGI1 Ab-mediated encephalitis diagnosis; OR 1.64; 95% CI 0.72–3.75; *p* = 0.244). In ordinal regression, with 0 representative of an mRS > 2, 1 an mRS < 3 combined with one of DRE or significant memory impairment, and 2 a favourable composite outcome, the associations all again remained similar compared to the original composite outcome model (Table S11).

## Discussion

This study outlines disease characteristics in a large cohort of Australian patients with AE and identifies prognostic features for functional and clinical outcomes. We also used a novel binary composite functional-clinical outcome measure—which combines mRS, DRE and memory impairment—as an additional assessment scale to improve disease monitoring. Younger age and anti-LGI1 Ab-mediated encephalitis were associated with better outcomes at 12 months, and RSE, acute T2/FLAIR hyperintensity on MRI, first-line immunotherapy failure and a diagnosis of definite AILE were associated with worse outcomes.

In patients with anti-NMDAR Ab-mediated encephalitis, age typically has not predicted functional outcomes [4], and results are mixed in patients with anti-LGI1 encephalitis [15, 36], though a study of antibody-negative cases found age more than 60 to be a marker of poor prognosis [5]. A systematic review found few studies reported association between age and outcome in AE [14]. It is possible that older individuals in some cohorts could have greater pre-morbid functional deficits than in others, and we were also unable to account for this in our cohort.

Our results suggest anti-LGI1 Ab-mediated encephalitis carries a better functional and clinical prognosis than other forms of AE. Most patients with anti-LGI Ab-mediated encephalitis achieve a “good” mRS of 2 or less over time, but residual memory deficit occurs in a proportion, particularly in verbal and visuospatial memory domains [15, 30]. Indeed, the odds of achieving a favourable composite outcome were lower than that of a favourable mRS, likely due to residual memory impairment. It should be noted that for prediction of a favourable mRS alone, the wide confidence interval suggests some uncertainty as to the exact effect size of an anti-LGI1 Ab-mediated encephalitis diagnosis in a broad cohort of AE patients. Furthermore in these patients, epilepsy is a recognised sequela, occurring in up to 20% of cases [37]. Therefore, mRS may not adequately reflect morbidity in patients with anti-LGI1 Ab-mediated encephalitis [8]. Other deficits observed after anti-LGI1 ab-mediated encephalitis include mood impairment and fatigue [8], therefore, our current outcome measures may not have reflected all possible morbidity in this population. Finally, in our cohort all patients with anti-LGI1 Ab-mediated encephalitis received at least first-line immunotherapy—a known modulator of long-term disability [15].

Conversely, we found definite AILE to associate with worse composite clinical-functional outcome at 12 months. Notably, DRE occurred in 31% and contrasts with a retrospective cohort of 12 antibody-negative LE in whom seizures where rarely seen [38], and another study which demonstrated 18 of 23 patients had a favourable mRS at 24 months [5]. Comparisons between these studies are limited due to different number of autoantibody test panels, and thus potential variability in exclusion of seropositive AE forms [6]. Nonetheless, it is possible a larger and potentially more heterogeneous patient population may be ascribed to this diagnosis. In regards to cognitive sequelae, we previously found that mesial temporal lobe T2/FLAIR hyperintensity on MRI are associated with later mesial temporal atrophy [33], and the latter can predict memory deficits [30]. Whether these findings are unique to antibody-negative limbic encephalitis warrants clarification with larger patient populations and more stringent inclusion criteria.

We found acute RSE to be a poor prognostic feature in our cohort. RSE in this population as an initial clinical feature is also referred to as new-onset refractory status epilepticus (NORSE) [28]. It has been shown previously that SE duration in NORSE predicts poor outcome (mRS > 3) [39]. The causes of NORSE in this study, however, were more heterogeneous than in our cohort, and included non-immune mediated aetiologies. Though other clinical features of AE or autoantibody results may not emerge in a timely enough manner to guide acute treatment decision making in patients with NORSE, our findings suggest the need to control seizures in patients with a suspected immune basis for SE. Larger studies are needed to evaluate the prognostic significance of RSE in specific AE-subtypes. Acute symptomatic seizures in AE are generally immunotherapy responsive [12, 15], and not typically associated with poorer functional outcomes [40], though few studies have explored this association.

Acute MRI T2/FLAIR hyperintensity was consistently associated with worse outcomes in our cohort. In anti-NMDAR Ab-mediated encephalitis, association between T2/FLAIR abnormalities and poor functional outcomes has been described in one study [17], but not others [40]. In patients with anti-LGI1 Ab-mediated encephalitis, mesial temporal lobe hyperintensity is known to be associated with cognitive impairment [15]. Given MRI abnormalities typically appear early in the disease, their presence could guide more aggressive immunotherapy. It is possible the prognostic significance of MR abnormalities varies between AE subtypes, and this warrants evaluation in future larger studies.

First-line immunotherapy failure predicting poor prognosis may suggest an early functional improvement represents an important treatment target in AE. The disproportionate use of second-line relative to first-line immunotherapy in our study may have influenced this finding, given efficacy of use of second-line immunotherapy has been shown in anti-NMDAR Ab-mediated encephalitis patients in this context [4]. Though our first-line failure definition included patients without mRS improvement prior to 2nd line immunotherapy before 4 weeks, we adjusted for initial disease severity in our multivariable model, which may otherwise have explained this occurrence.

We found no association between routine FBE and CSF parameters and outcomes. In anti-NMDAR Ab-mediated encephalitis, multiple cohorts have shown an association between CSF pleocytosis and poor outcome [4], but it is possible a larger cohort than ours is required to evaluate this effect. The presence of an inflammatory CSF profile varies across different autoimmune encephalitides [41], therefore, such changes could have differing significance across subtypes. Indeed, CSF is commonly bland in some antibody-mediated syndromes [41]. Further, we only included the initial CSF for analysis, which, alongside peripheral immune ratios, may vary over time in a given patient. Nonetheless, we believe peripheral blood immune cell counts and CSF findings are unlikely to inform long-term outcomes.

There is emerging recognition of the limitations of the mRS and the need for more targeted and disease specific outcome measures in AE [7, 8, 37]. In our cohort, 35 (16%) patients with a “favourable” mRS had either drug-resistant epilepsy or significant memory impairment at 12 months. While further recovery may occur after 12 months in some patients—particularly a subset of those with anti-NMDAR Ab-mediated encephalitis [11]—our results reinforce the need for disease-specific severity measures in future studies. Our sensitivity analyses revealed that altering the constitution of the composite outcome did not considerably alter its associations with underlying covariates in all but one model. These findings suggest that the composite outcome is relatively robust and not overly dependent on a single component.

### Limitations

Our study has several limitations. The predominantly retrospective manner of data collection impacted consistency of relevant clinical information, particularly for serial assessments. As indicated, an mRS of ≤ 2 as a favourable outcome may be insensitive to the spectrum of morbidity remaining in certain patients. Despite our use of a composite clinical-functional outcome measure, we could not consistently assess other relevant clinical and functional endpoints, such as fatigue, sleep and mood disturbance, and specific cognitive sequelae, which may have had particular relevance to patients with anti-LGI1 Ab-mediated encephalitis. Owing to the rarity of AE, we combined different forms of the disease. However, inherent differences exist with respect to natural history, clinical and epidemiological features, particularly with respect to seronegative patients. We adjusted for this by inclusion of common diagnostic categories as covariates in our analyses, but differences between subtypes could still lead to residual confounding.

Variability in antibody-testing availability over time and lack of local access to testing for rarer forms meant it was not possible to uniformly classify relevant patients as ‘seronegative.’ While fulfilment of ‘possible’ criteria had been intended as means for consideration of evaluation for AE rather than representing a final diagnosis, such patients included in our cohort had no better explanation for their clinical presentation on specific review of available medical records. A recent study found of 107 patients misdiagnosed with AE, 77 (72%) did not fulfil the two initial requirements of the ‘possible’ criteria [3]. Our final cohort consisted of patients who fulfilled the first two criteria (subacute onset of working memory deficits, altered mental status or psychiatric symptoms—with addition of patients with broader cognitive syndromes such as confusion—and at least one of new focal CNS findings, de novo seizures, CSF pleocytosis and compatible MRI features), and in whom no better explanation could be provided with regards to the third (reasonable exclusion of alternative causes). Exclusion of patients who died of causes deemed not directly related to the underlying AE or associated malignancy may have underestimated the overall burden of this group of diseases, but this only consisted of five patients.

Furthermore, comparatively less frequent use of second-line immunotherapy and likely indication bias prevented meaningful analysis of its efficacy. Finally, we did not collect data on treatment-related adverse effects, which must be considered in clinical decision making.

## Conclusion

This study details a large cohort of patients with AE and reports functional and clinical outcomes in this group. Older age, MRI/T2 FLAIR hyperintensity, RSE and first-line immunotherapy failure predict patients less likely to achieve favourable functional outcome at 12 months. A diagnosis of anti-LGI1 Ab-mediated encephalitis when compared to all other patients with AE predicted a better twelve-month functional outcome. Our findings may inform clinical decisions in the acute disease phase in particular, and warrant exploration in future prospective studies, with focus on combined and comprehensive functional and clinical outcomes.

## Supplementary Information

Below is the link to the electronic supplementary material.

Supplementary file 1 (DOCX 39 KB)

## Data Availability

Anonymised data may be shared to any qualified investigators by the corresponding author upon reasonable requests.
