# Supplementary material for: Multimodal prognostication of autoimmune encephalitis: an Australian autoimmune encephalitis consortium study
Source: J Neurol. 2025 Apr 25;272(5):361. doi: 10.1007/s00415-025-13069-1 (PMC12031909; doi:10.1007/s00415-025-13069-1)
Supplement: Supplementary file 1 — (DOCX 39 KB) [file 415_2025_13069_MOESM1_ESM.docx]

**Multimodal Prognostication of Autoimmune Encephalitis:** Supplementary appendix

**Table S1** Associated malignancies active at time of diagnosis or detected subsequent to AE diagnosis

|  | All | Possible^a^ | Probable^a^ | AILE^a^ | NMDAR^a^ | LGI1 | CASPR2 | GABAB | AMPAR | IgLON5 | GFAP | MOG-CE^b^ | ICI-E |
| --- | --- | --- | --- | --- | --- | --- | --- | --- | --- | --- | --- | --- | --- |
| Tumour | 33 (14%) | 9 (10%) | 2 (18%) | 3 (19%) | 13 (27%) | 0 (0%) | 0 (0%) | 1 (50%) | 2 (50%) | 0 (0%) | 0 (0%) | 0 (0%) | 3 (100%) |
| Type |  |  |  |  |  |  |  |  |  |  |  |  |  |
| Dermoid | 11 | 1 | 0 | 0 | 10 | 0 | 0 | 0 | 0 | 0 | 0 | 0 | 0 |
| SCLC | 2 | 2 | 0 | 0 | 0 | 0 | 0 | 0 | 0 | 0 | 0 | 0 | 0 |
| IDC breast | 2 | 2 | 0 | 0 | 0 | 0 | 0 | 0 | 0 | 0 | 0 | 0 | 0 |
| Thymoma | 2 | 0 | 0 | 1 | 0 | 0 | 0 | 0 | 1 | 0 | 0 | 0 | 0 |
| Lung SCC | 1 | 0 | 0 | 0 | 0 | 0 | 0 | 0 | 0 | 0 | 0 | 0 | 1 |
| Breast cancer | 2 | 1 | 0 | 1 | 0 | 0 | 0 | 0 | 0 | 0 | 0 | 0 | 0 |
| Thymic carcinoid | 1 | 1 | 0 | 0 | 0 | 0 | 0 | 0 | 0 | 0 | 0 | 0 | 0 |
| SCC | 1 | 0 | 0 | 0 | 0 | 0 | 0 | 0 | 0 | 0 | 0 | 0 | 1 |
| Ewing’s | 1 | 0 | 0 | 0 | 0 | 0 | 0 | 0 | 1 | 0 | 0 | 0 | 0 |
| Ovarian fibrothecoma | 1 | 0 | 0 | 0 | 1 | 0 | 0 | 0 | 0 | 0 | 0 | 0 | 0 |
| Colorectal ca | 1 | 1 | 0 | 0 | 0 | 0 | 0 | 0 | 0 | 0 | 0 | 0 | 0 |
| Renal cell ca. | 1 | 0 | 0 | 0 | 0 | 0 | 0 | 0 | 0 | 0 | 0 | 0 | 1 |
| Hodgkin’s lymphoma | 1 | 0 | 0 | 1 | 0 | 0 | 0 | 0 | 0 | 0 | 0 | 0 | 0 |
| Large cell neuroendocrine | 1 | 0 | 0 | 0 | 1 | 0 | 0 | 0 | 0 | 0 | 0 | 0 | 0 |
| CLL | 1 | 0 | 1 | 0 | 0 | 0 | 0 | 0 | 0 | 0 | 0 | 0 | 0 |
| Suspected lung ca. on CT | 2 | 1 | 0 | 0 | 0 | 0 | 0 | 1 | 0 | 0 | 0 | 0 | 0 |
| Ovarian mass, pathology inconclusive | 1 | 0 | 0 | 0 | 1 | 0 | 0 | 0 | 0 | 0 | 0 | 0 | 0 |
| Submandibular mass | 1 | 0 | 1 | 0 | 0 | 0 | 0 | 0 | 0 | 0 | 0 | 0 | 0 |

**Table S2** Causes of death prior to 12 months

| Subject | Cause of death | Inclusion in models |
| --- | --- | --- |
| 1 | Poor neurological outcome - palliated | Y |
| 2 | Progressive neurologic disease | Y |
| 3 | Suicide in context of AE | Y |
| 4 | Progressive neurologic disease | Y |
| 5 | Super refractory status epilepticus - palliated | Y |
| 6 | Pneumonia | N |
| 7 | STEMI | N |
| 8 | Urosepsis | N |
| 9 | Poor neurological outcome | Y |
| 10 | PE | N |
| 11 | Poor neurological outcome - palliated | Y |
| 12 | Aspiration pneumonitis | N |
| 13 | Status epilepticus | Y |
| 14 | Poor neurological outcome - palliated | Y |
| 15 | Metastatic cancer - palliated | Y |
| 16 | Progressive neurologic disease | Y |
| 17 | Malignancy | Y |
| 18 | Intracranial haemorrhage into CNS-disease related lesions | Y |

**Table S3**

Univariable analyses of clinical variables in relation to outcome of favourable 12 month mRS and favourable 12 month composite status

|  | 12 months favourable mRS | | 12 months favourable composite outcome^a^ | |
| --- | --- | --- | --- | --- |
|  | OR (95% CI) | p | OR (95% CI) | p |
| Age at onset | **0.97 (0.96, 0.99)** | **<0.001** | **0.98 (0.97, 0.99)** | **0.002** |
| Sex (male) | 1.30 (0.75, 2.25) | 0.35 | 1.14 (0.89, 1.91) | 0.63 |
| Possible AE | 0.90 (0.51, 1.57) | 0.71 | 1.17 (0.69, 1.99) | 0.56 |
| Probable AE | **0.27 (0.08, 0.94)** | **0.04** | 0.34 (0.09, 1.30) | 0.12 |
| Definite AILE | **0.20 (0.07, 0.60)** | **0.005** | **0.05 (0.01, 0.42)** | **0.006** |
| NMDAR | 1.71 (0.84, 3.51) | 0.14 | 1.65 (0.87, 3.14) | 0.13 |
| LGI1 | **2.62 (1.10, 6.25)** | **0.03** | **2.12 (1.03, 4.39)** | **0.04** |
| Initial mRS | **0.69 (0.54, 0.90)** | **0.007** | **0.75 (0.59, 0.96)** | **0.02** |
| Initial CASE | **0.94 (0.89, 0.99)** | **0.03** | **0.95 (0.89, 1.00)** | **0.06** |
| Mechanical ventilation | **0.47 (0.25, 0.86)** | **0.02** | **0.52 (0.29, 0.97)** | **0.04** |
| Tumour | 0.63 (0.30, 1.34) | 0.24 | 0.56 (0.27, 1.20) | 0.14 |
| Seizure | 1.06 (0.60, 1.88) | 0.85 | 0.84 (0.49, 1.44) | 0.52 |
| SE | **0.45 (0.23, 0.89)** | **0.02** | **0.32 (0.15, 0.67)** | **0.003** |
| RSE | **0.22 (0.09, 0.55)** | **0.001** | **0.12 (0.03, 0.40)** | **0.001** |
| Conscious state | 0.71 (0.41, 1.23) | 0.23 | 0.75 (0.45, 1.27) | 0.29 |
| Psychiatric^b^ | **1.94 (1.10, 3.40)** | **0.02** | 1.60 (0.93, 2.75) | 0.09 |
| Neutrophils | 0.99 (0.92, 1.06) | 0.74 | 1.00 (0.93, 1.07) | 0.99 |
| Lymphocytes | 1.00 (0.75, 1.33) | 1.00 | 1.02 (0.78, 1.34) | 0.87 |
| Monocytes | 0.84 (0.41, 1.69) | 0.62 | 0.73 (0.37, 1.44) | 0.37 |
| NLR | 1.00 (0.94, 1.06) | 0.88 | 0.97 (0.92, 1.03) | 0.39 |
| MLR | 0.62 (0.26, 1.51) | 0.30 | 0.48 (0.20, 1.15) | 0.10 |
| CSF pleocytosis^c^ | 1.10 (0.63, 1.91) | 0.75 | 1.00 (0.95, 1.06) | 0.88 |
| CSF WCC > 20^c^ | 1.36 (0.70, 2.65) | 0.36 | 1.09 (0.61, 2.00) | 0.78 |
| CSF lymphocytes | 1.00 (1.00, 1.01) | 0.26 | 1.00 (1.00, 1.00) | 0.87 |
| CSF total protein | 1.03 (0.60, 1.78) | 0.91 | 0.94 (0.56, 1.58) | 0.78 |
| MR T2/FLAIR abnormality | **0.26 (0.15, 0.47)** | **<0.001** | **0.30 (0.17, 0.54)** | **<0.001** |
| EEG focal slow | 0.92 (0.49, 1.92) | 0.80 | 0.69 (0.38, 1.27) | 0.24 |
| EEG general slow | **0.47 (0.26, 0.82)** | **0.01** | **0.53 (0.31, 0.91)** | **0.02** |
| EEG discharges | 0.76 (0.38, 1.54) | 0.46 | 0.81 (0.41, 1.59) | 0.53 |
| EEG seizures | 0.63 (0.31, 1.30) | 0.21 | 0.70 (0.34, 1.46) | 0.35 |
| Relapse^d^ | **3.68 (1.06, 12.80)** | **0.04** | 1.88 (0.76, 4.61) | 0.17 |
| Treatment delay^e^ | 1.08 (0.62, 1.89) | 0.78 | 1.07 (0.63, 1.81) | 0.81 |
| First line failure | **0.18 (0.10, 0.34)** | **<0.001** | **0.22(0.13, 0.38)** | **<0.001** |
| Second line < 12m | 0.75 (0.42, 1.33) | 0.32 | 0.72 (0.41, 1.25) | 0.25 |

^a^Composite of mRS < 2, absence of DRE and significant cognitive impairment

^b^Delusions, hallucinations, behavioural disturbance, mood disturbance, thought disorder

^c^Dichotomous variable

^d^Within 12 months

^f^Treatment delay > 28 days, untreated patients assigned as treatment delay

mRS, modified Rankin scale; AILE, autoimmune limbic encephalitis; NMDAR, *N*-methyl-D-aspartate receptor; LGI1, leucine-rich glioma-inactivated 1; SE, status epilepticus; RSE, refractory status epilepticus; FLAIR, fluid attenuated inversion recovery; EEG, electroencephalography; IVIg, intravenous immunoglobulin

**Table S4** Multivariable logistic regression prior to backward elimination (elimination based on largest p-values), for favourable mRS at 12m

|  | *p* | OR (95% CI) |
| --- | --- | --- |
| Age onset | **0.001** | **0.97 (0.95, 0.98)** |
| Probable | 0.58 | 0.62 (0.11, 3.43) |
| AILE | 0.05 | 0.22 (0.05, 1.01) |
| LGI1 | 0.04 | 3.31 (1.09, 10.10) |
| Initial mRS | 0.16 | 0.77 (0.54, 1.10) |
| Mechanical ventilation | 0.78 | 1.16 (0.42, 3.17) |
| Psychiatric | 0.28 | 1.52 (0.71, 3.27) |
| Refractory SE | 0.02 | 0.21 (0.06, 0.82) |
| MRI T2/FLAIR | **0.009** | **0.36 (0.17, 0.77)** |
| EEG generalised slowing | 0.57 | 0.77 (0.32, 1.87) |
| First line failure | **<0.001** | **0.16 (0.07, 0.34)** |
| Relapse | 0.15 | 3.12 (0.67, 14.65) |

AILE: autoimmune limbic encephalitis; LGI1, leucine-rich glioma-inactivated 1; mRS, modified Rankin scale; SE, status epilepticus; FLAIR, fluid attenuated inversion recovery; EEG, electroencephalography

**Table S5** Bootstrapped estimated multivariable logistic regression for favourable mRS

| **Variable** | **OR (95% CI)** |
| --- | --- |
| Age onset | 0.97 (0.95, 0.98) |
| First-line failure | 0.15 (0.06, 0.33) |
| MRI T2-FLAIR | 0.24 (0.11, 0.52) |
| Refractory SE | 0.16 (0.05, 0.50) |
| Anti-LGI1 | 5.11 (1.73, 19.40) |

FLAIR, fluid attenuated inversion recovery; SE, status epilepticus; AILE: autoimmune limbic encephalitis; LGI1, leucine-rich glioma-inactivated 1

**Table S6** Multivariable logistic regression model for composite outcome pre-backward selection

|  | *p* | OR (95% CI) |
| --- | --- | --- |
| Age onset | **<0.001** | **0.97 (0.95, 0.99)** |
| AILE | **0.007** | **0.04 (0.005, 0.44)** |
| LGI1 | **0.04** | **2.67 (1.07, 6.67)** |
| Initial mRS | 0.41 | 0.87 (0.62, 1.22) |
| Mechanical ventilation | 0.48 | 1.39 (0.56, 3.47) |
| Refractory SE | **0.001** | **0.08 (0.02, 0.35)** |
| EEG generalised slowing | 0.33 | 0.69 (0.32, 1.46) |
| MRI T2/FLAIR | **0.02** | **0.42 (0.20, 0.84)** |
| First-line failure | **<0.001** | **0.18 (0.09, 0.35)** |

AILE: autoimmune limbic encephalitis; LGI1, leucine-rich glioma-inactivated 1; mRS, modified Rankin scale; SE, status epilepticus; EEG, electroencephalography; FLAIR, fluid attenuated inversion recovery

**Table S7** Bootstrapped estimated multivariable logistic regression for favourable composite outcome

| **Variable** | **OR** | **95% CI** |
| --- | --- | --- |
| Age onset | 0.969 | 0.951 - 0.987 |
| First-line failure | 0.201 | 0.096 - 0.393 |
| MRI T2-FLAIR | 0.404 | 0.188 - 0.816 |
| Refractory SE | 0.030 | 0.000 - 0.210 |
| Anti-LGI1 | 2.626 | 1.014 - 7.675 |
| AILE | 0.000 | 0.000 - 0.263 |

FLAIR, fluid attenuated inversion recovery; SE, status epilepticus; AILE: autoimmune limbic encephalitis; LGI1, leucine-rich glioma-inactivated 1; AILE: autoimmune limbic encephalitis

**Table S8** Multivariable logistic regression for predictors of composite outcome consisting of a favourable mRS and absence of significant memory impairment

| **Variable** | **OR** | **95% CI** | **p-value** |
| --- | --- | --- | --- |
| Age onset | 0.97 | 0.95 - 0.99 | <0.001 |
| First-line failure | 0.21 | 0.11 - 0.39 | <0.001 |
| MRI T2/FLAIR | 0.42 | 0.21 - 0.84 | 0.015 |
| Refractory SE | 0.11 | 0.03 - 0.39 | 0.001 |
| Anti-LGI1 | 2.47 | 1.02 - 5.98 | 0.047 |
| AILE | 0.20 | 0.04 - 0.95 | 0.044 |

mRS, modified Rankin scale; FLAIR, fluid attenuated inversion recovery; SE, status epilepticus; LGI1, leucine-rich glioma-inactivated 1; AILE, autoimmune limbic encephalitis

**Table S9** Multivariable logistic regression for predictors of composite outcome consisting of a favourable mRS and absence of drug-resistant epilepsy

| **Variable** | **OR** | **95% CI** | **p-value** |
| --- | --- | --- | --- |
| Age at onset | 0.97 | (0.95, 0.99) | 0.002 |
| First-line failure | 0.18 | (0.08, 0.41) | < 0.001 |
| MRI T2 FLAIR | 0.37 | (0.18, 0.79) | 0.011 |
| Refractory SE | 0.09 | (0.03, 0.31) | < 0.001 |
| LGI1 | 3.90 | (1.38, 11.05) | 0.011 |
| AILE | 0.06 | (0.01, 0.38) | 0.003 |

mRS, modified Rankin scale; FLAIR, fluid attenuated inversion recovery; SE, status epilepticus; LGI1, leucine-rich glioma-inactivated 1; AILE, autoimmune limbic encephalitis

**Table S10** Multivariable logistic regression for predictors of composite outcome consisting of a absence of significant memory impairment and absence of drug-resistant epilepsy

| **Variable** | **OR** | **95% CI** | **p-value** |
| --- | --- | --- | --- |
| Age at onset | 0.98 | 0.96 - 0.99 | 0.003 |
| First-line failure | 0.29 | 0.15 - 0.54 | <0.001 |
| MRI T2 FLAIR | 0.51 | 0.27 - 0.99 | 0.049 |
| Refractory SE | 0.18 | 0.06 - 0.55 | 0.003 |
| LGI1 | 1.64 | 0.72 - 3.75 | 0.244 |
| AILE | 0.16 | 0.04 - 0.70 | 0.016 |

mRS, modified Rankin scale; FLAIR, fluid attenuated inversion recovery; SE, status epilepticus; LGI1, leucine-rich glioma-inactivated 1; AILE, autoimmune limbic encephalitis

**Table S11** Multivariable ordinal regression for predictors of weighted composite outcome*

| **Predictor** | **OR** | **95% CI** | ***p*** |
| --- | --- | --- | --- |
| 0\|1 | 0.03 | 0.01-0.07 | <0.001 |
| 1\|2 | 0.06 | 0.02-0.16 | <0.001 |
| Age onset | 0.97 | 0.96 - 0.99 | <0.001 |
| AILE | 0.19 | 0.05 - 0.69 | 0.012 |
| LGI11 | 2.66 | 1.17 - 6.03 | 0.020 |
| Refractory SE | 0.15 | 0.06 - 0.42 | <0.001 |
| MRI T2/FLAIR | 0.41 | 0.22 - 0.79 | 0.008 |
| First-line failure | 0.21 | 0.11 - 0.39 | <0.001 |

* mRS > 2 = 0, mRS < 3 and one of poor memory or DRE = 1, mRS < 3 and no poor memory or DRE = 2.

AILE, autoimmune limbic encephalitis; LGI1, leucine-rich glioma-inactivated 1; SE, status epilepticus; FLAIR, fluid attenuated inversion recovery; mRS, modified Rankin scale
